# Effectiveness of e-Health cardiac rehabilitation program on quality of life associated with symptoms of anxiety and depression in moderate-risk patients

**DOI:** 10.1038/s41598-021-83231-y

**Published:** 2021-02-12

**Authors:** Raquel Bravo-Escobar, Alicia González-Represas, Adela María Gómez-González, Ángela Heredia-Torres

**Affiliations:** 1 Cardiac Rehabilitation Unit, Virgen de La Victoria University Hospital of Malaga, Campus de Teatinos s/n, 29010 Malaga, Spain; 2 Department of Functional Biology and Health Sciences, Physical Therapy Faculty, University of Vigo, Campus A Xunqueira s/n, 36005 Pontevedra, Spain; 3 Cardiac Rehabilitation Unit, Reina Sofía University Hospital of Córdoba, Avda. Menéndez Pidal, s/n, 14004 Córdoba, Spain

**Keywords:** Cardiology, Health care

## Abstract

Exploring new models of medical care requires evaluating the impact of new care strategies not only on physiological parameters but also on the quality of life of the patient. On the other hand the presence of anxiety together with depression requires further consideration when planning appropriate management strategies. The aim of this study was to examine the effectiveness of a home-based cardiac rehabilitation program incorporating an e-Health technology on health-related quality of life associated with symptoms of anxiety and depression in moderate-risk patients. A multicenter, randomized controlled clinical trial was designed to compare a traditional hospital based cardiac rehabilitation program (n = 38, 35 male) with a mixed home surveillance program where patients exercised at home with a remote electrocardiographic monitoring device (n = 33, 31 male). The Short Form-36 (SF-36) Health Survey and the Goldberg questionnaire were used to evaluate quality of life and the presence of symptoms of anxiety and depression respectively. The results of this study show that the type of cardiac rehabilitation program did not influence the improvement in quality of life (p = 0.854), but the presence of symptoms of anxiety and depression did (p = 0.001). Although both programs achieved a decrease in anxiety and depression symptoms and improved functional capacity (p ≤ 0.001), a significant interaction effect was found between the group with or without anxiety and depression symptoms and the type of program in the bodily pain dimension (p = 0.021). *Trial registration*: Retrospectively registered NCT02796404 (10/06/2016) in clinialtrials.gov.

## Introduction

In patients with cardiovascular disease (CVD), depression and anxiety interfere with the recovery process and are associated not only with a poorer prognosis and increased long-term mortality^1–3^, but also with reduced productivity due to significantly increased disability rates in the working population and increased medical care costs^4–6^. However, despite the serious impact of depression and anxiety, these disorders are often not detected or not properly addressed^7,8^.

The presence of anxiety together with depression requires further consideration when planning appropriate management strategies^9^. Early detection of anxiety and depression symptoms in patients with coronary heart disease starting cardiac rehabilitation programs may translate into improved healthcare models^10^. The evaluation of the efficacy of new cardiac rehabilitation programs should be carried out according to the presence or absence of these symptoms since they may involve modifications in their design.

Currently, most cardiac rehabilitation programs are conducted in hospitals or outpatient centers, and while there is strong evidence of benefits in terms of functional capacity, quality of life and patient prognosis, only 10–30% of eligible patients access these programs^11–14^. In recent years, different models of home-based cardiac rehabilitation have been developed to expand coverage and accessibility to these programs without increasing total costs^15^. Although most of the programs have been implemented in low-risk patients^16^, the current situation is accelerating the transformation of the healthcare model and advances in technology now make it possible to develop home-based cardiac rehabilitation programs for higher-risk patients^17^ who require a higher degree of monitoring or supervision^16^.

Evaluating the effectiveness of new health care models must consider not only physiological parameters changes but also changes in quality of life. To our knowledge no studies have analyzed the effectiveness of these programs on quality of life associated with the presence or absence of anxiety and depression in moderate-risk patients, even though they are predictors of quality of life^18^. It is therefore important to consider these symptoms when assessing the effectiveness of new models of cardiac rehabilitation care.

The aim of this study was to examine the effectiveness of a home-based cardiac rehabilitation program using mixed monitoring on health-related quality of life compared with hospital-based cardiac rehabilitation programs, considering symptoms of anxiety and depression.

## Results

A total of 80 patients took part in the study, of whom 40 participated in the standard hospital-based cardiac rehabilitation program (control group) and 40 in the home-based cardiac rehabilitation program with mixed monitoring (experimental group). Patients with symptoms of anxiety only or depression only were excluded from the analysis due to the limited number of cases (9). The final sample comprised 71 patients, 38 in the control group and 33 in the experimental group. The program follow-up in both cases was complete.

The mean age of the patients was 55.72 ± 8.23 years (age range 36–75 years) with 93% being men. Most patients underwent percutaneous transluminal coronary angioplasty with pharmacological stenting (85.9%). The median time to start the cardiac rehabilitation program was 120 days (IQR 101) from the surgery with no significant differences between control and experimental group (p = 0.113).

Of the study sample, 64.78% (46) displayed symptoms of both anxiety and depression according to the Goldberg score (24 subjects in the hospital program and 22 in the home program) and a poorer quality of life that primarily affected the dimensions of physical role (27.81 ± 37.59 vs 61 ± 43.94) and emotional role (32.95 ± 43.73 vs 84 ± 37.41). Although the anxiety and depression scores at the start of the study in both programs were high, the participants in the home program had a higher anxiety score (Table 1).

**Table 1:** Baseline characteristics of the sample.

	Hospital (n = 38)	Home (n = 33)	p-value
Age (years)	55.32 (7.97)	56.18 (8.71)	ns
Sex, male n (%)	35 (92.10)	31 (93.93)	ns
**Risk factors**			
Hypertension n (%)	23 (50.52)	18 (54.54)	ns
Hyperlipidemia n (%)	16 (42.10)	17 (51.51)	ns
Diabetes n (%)	6 (15.7)	12 (36.36)	ns
Overweight n (%)	19 (50)	19 (57.57)	ns
Obese n (%)	12 (31.57)	9 (27.27)	ns
BMI (kg/m^2^)	29.01 (3.48)	28.47 (3.67)	ns
Waist-hip ratio (cm)	104.28 (9.07)	102.62 (9.71)	ns
Current smokers n (%)	4 (10.52)	2 (6.06)	ns
**Revascularization technique**			
CABG n (%)	3 (7.89)	4 (12.12)	ns
PCI n (%)	34 (89.47)	27 (81.81)	ns
PCI & CABG n (%)	1 (2.63)	2 (6.06)	ns
**Blood pressure**			
Systolic blood pressure (mmHg)	122.68 (22.82)	122.48 (23.31)	ns
Diastolic blood pressure (mmHg)	73.28 (11.03)	74.69 (11.47)	ns
**Cardiopulmonary exercise test**			
Exercise time (min)	7 (2.05)	6.43 (2.65)	ns
METS	8.77 (2.39)	8.23 (2.71)	ns
Hypertension response n (%)	10 (26.31)	5 (15.5)	ns
Positive clinical response n (%)	0 (0)	0 (0)	ns
Positive electrical response n (%)	4 (10.52)	0 (0)	ns
**Laboratory values**			
Total cholesterol (mg/dl)	139.68 (35.53)	140.81 (35.80)	ns
HDL cholesterol (mg/dl)	42.42 (12.26)	41.87 (9.71)	ns
LDL cholesterol (mg/dl)	72.28 (27.74)	70.72 (27.71)	ns
Triglycerides (mg/dl)	127.54 (52.85)	138.21 (62.72)	ns
Glucose (mg/dl)	102.32 (19.49)	105.96 (20.26)	ns
HbA1c	5.81 (0.64)	6.28 (1.45)	ns
**Health-related quality of life**			
SF-36 (score)	53.88 (20.60)	50.16 (24.01)	ns
Anxiety and Depression patients n (%)	24 (63.15)	22 (66.66)	ns
**Goldberg scale (score)**^**†**^			
Anxiety	6.25 (1.53)	7.31 (1.24)	0.014*
Depression	5.37 (2.28)	5.59 (1.89)	ns

Data are presented as mean values (SD).

*Significance level p < 0.05. Significant values are highlighted in bold.

^†^Score of patients with symptoms of depression and anxiety.

According to the results obtained (Table 2) the changes in quality of life did not depend on the type of program (p = 0.854) but on the presence or absence of symptoms of anxiety and depression (p = 0.001). In general, patients with these symptoms experienced a greater change in all dimensions of quality of life, with the exception of the bodily pain dimension and health transition (p = 0.784). All the patients who participated in the home-based or hospital-based cardiac rehabilitation program reported significant improvements in their health status, with the home-based cardiac rehabilitation program being as effective as the hospital-based program in improving functional capacity (p ≤ 0.001), regardless of symptoms of anxiety and depression. However a significant interaction effect was found between the group with or without symptoms of anxiety and depression and the type of program in the bodily pain dimension (p = 0.021). The perception of pain tended to decrease in patients who participated in the hospital-based cardiac rehabilitation program, while it increased in the home-based cardiac rehabilitation program, especially in those subjects who had anxiety and depression, although both programs achieved a significant decrease in these symptoms.

**Table 2:** Results obtained in quality of life, levels of anxiety and depression and physical capacity before and after the two different cardiac rehabilitation programs considering symptoms of anxiety and depression.

	Hospital-based cardiac rehabilitation program (n = 38)				Home-based cardiac rehabilitation program (n = 33)				p-value^a^	p-value^b^	p-value^c^	p-value^d^
	Anxiety and Depression (n = 24)		No anxiety and depression (n = 14)		Anxiety and depression (n = 22)		No anxiety and depression (n = 11)					
	Before	After	Before	After	Before	After	Before	After				
SF-36 (score)	44.08 (16.04)	49.39 (19.31)	70.64 (16.48)	72.71 (21.00)	40.25 (18.24)	44.91 (23.79)	69.98 (22.33)	76.61 (22.88)	0.006*	0.854	0.001*	0.273
Physical functioning	67.12 (19.83)	70.62 (20.34)	78.21 (19.96)	80.71 (15.54)	60.22 (21.07)	62.72 (23.79)	83.18 (11.24)	88.63 (9.24)	0.074	0.973	0.016*	0.286
Physical role functioning	18.75 (31.49)	31.25 (43.76)	51.78 (44.35)	71.42 (46.88)	22.72 (30.77)	26.13 (37.38)	72.72 (42.50)	68.18 (46.22)	0.064	0.179	0.005*	0.810
Bodily pain	59.83 (28.28)	59.12 (21.94)	76.32 (21.44)	73.75 (27.20)	43.50 (21.27)	47.36 (28.34)	76.36 (21.91)	78.45 (29.46)	0.605	0.199	0.001*	0.021*
General health perception	43.54 (12.46)	46.45 (20.34)	63.21 (19.17)	58.57 (22.73)	41.40 (18.30)	42.72 (20.27)	52.72 (23.59)	64.09 (17.72)	0.347	0.602	0.012*	0.417
Vitality	41.25 (20.01)	44.37 (18.95)	68.21 (18.35)	64.64 (26.63)	37.50 (19.62)	39.09 (25.80)	64.09 (22.34)	69.54 (24.74)	0.409	0.687	0.001*	0.135
Social role functioning	29.02 (23.27)	51.62 (28.22)	87.53 (15.38)	86.96 (20.82)	52.25 (23.76)	53.45 (31.88)	78.63 (25.99)	86.59 (27.11)	0.373	0.965	0.001*	0.341
Emotional role functioning	31.48 (44.17)	35.64 (47.13)	85.71 (36.31)	88.09 (30.96)	21.22 (39.39)	39.39 (48.94)	81.81 (40.45)	90.90 (30.15)	0.027*	0.975	0.001*	0.212
Mental health	50.37 (21.72)	53.66 (20.18)	76.57 (13.84)	80.28 (15.80)	44.97 (21.84)	49.09 (25.42)	77.45 (21.92)	81.45 (26.24)	0.161	0.979	0.001*	0.171
Health transition	34.37 (28.37)	52.08 (27.50)	44.64 (28.04)	50.00 (32.52)	38.63 (32.48)	44.18 (35.17)	43.18 (29.77)	70.45 (33.20	0.001*	0.090	0.784	0.084
**Goldberg scale**												
Anxiety	6.25 (1.53)	4.62 (2.85)	0.50 (0.85)	0.87 (1.51)	7.31 (1.24)	5.36 (2.23)	0.90 (1.22)	0.45 (0.68)	0.000*	0.839	0.001*	0.082
Depression	5.37 (2.28)	3.50 (2.75)	0.28 (0.46)	0.57 (0.93)	5.59 (1.89)	3.72 (2.37)	0.18 (0.40)	0.36 (1.20)	0.001*	0.284	0.001*	0.345
**Exercise capacity**												
METs	8.80 (2.47)	10.51 (2.95)	8.72 (2.34)	9.87 (2.42)	7.95 (2.93)	9.74 (3.34)	8.78 (2.24)	11.29 (2.66)	0.001*	0.166	0.269	0.095
Exercise time	7.09 (2.09)	7.96 (2.55)	6.85 (2.04)	7.74 (1.87)	6.16 (2.93)	7.65 (2.85)	6.98 (2.00)	9.20 (2.41)	0.000*	0.422	0.101	0.595

Data are presented as mean values (SD).

*Significance level p < 0.05. Significant values are highlighted in bold.

^a^Significance level for the hypothesis of no time effect.

^b^Significance level for the hypothesis of no time x program effect.

^c^Significance level for the hypothesis of no time x group effect.

^d^Significance level for the hypothesis of no time x group x program effect.

## Discussion

Until now, no studies have analyzed the effectiveness of a home-based cardiac rehabilitation program on quality of life in patients with coronary heart disease and moderate risk, taking into consideration symptoms of anxiety and depression, despite their high prevalence. Although patients with coronary heart disease have decreased quality of life^32,33^, according to the results of this study, the deterioration is significantly greater when these symptoms are present, especially in physical and emotional role functioning.

The home-based cardiac rehabilitation program was as effective as the hospital-based program in improving anxiety and depression symptoms, functional capacity and quality of life. Exercise together with educational and psychological interventions in hospital-based cardiac rehabilitation programs had a positive impact on the psychological state of the patients^10,34,35^. All these factors were also present in the home-based cardiac rehabilitation program with mixed monitoring, which was designed to allow these patients to receive psychological counseling and health education in group sessions and to maintain close contact with the different professionals of the multidisciplinary cardiac rehabilitation program team. The use of an e-Health support system in both programs may also have provided greater assurance to the patients and helped to reduce the levels of anxiety and depression. In fact, Giallura et al.^36^ argue that the use of an ECG monitoring device for recording exercise sessions appears to have a positive effect in these patients. In their study, significant improvements were found in anxiety and depression in a home-based cardiac rehabilitation program that used an ECG device. These results were comparable to those achieved with a hospital-based program, while the home-based group that did not use a device showed no improvement in either disorder.

Although the increase in physical capacity in moderate-risk patients in the home-based cardiac rehabilitation program was comparable to the increase achieved in the hospital-based program, the improvements in quality of life focused only on emotional role and health transition. Overall, the studies published to date have found improvements in virtually all the quality of life dimensions with no differences between the two programs. However, the patients included in the studies were low risk^37–39^ or low to moderate risk^40,41^, and the duration of the programs in most was 3 months^40,41^ to 6 months^42,43^.

Kraal et al.^40^ found a significant improvement in the physical, emotional and social dimensions of quality of life in both the hospital- and the home-based cardiac rehabilitation programs in patients with low to moderate risk, while Ades et al.^41^ observed an improvement in quality of life in both programs and in all dimensions (physical functioning, physical and emotional role limitations, social functioning, bodily pain and energy/fatigue), except mental health and health perception, using an electrocardiogram and online voice transtelephonic monitoring. Smith et al.^42^ and Arthur et al.^43^, however, found differences in observed changes in quality of life between hospital- and home-based cardiac rehabilitation programs. While both programs improved quality of life, the home-based group achieved significantly greater improvement than the hospital-based group in the physical^42,43^ and mental^42^ components. In both cases, the duration of the programs was 6 months and home-based patients reported significantly higher levels of social support compared with the hospital-based patients.

It is possible that the length of both programs in our study was not sufficient to achieve a greater effect on quality of life in moderate-risk patients. Nonetheless, a significant improvement was found. In fact, the SF-36 health transition question, which determines the magnitude of the minimal clinically important change and allows us to distinguish those patients who improve from those who deteriorate^44^, showed a significant improvement in health, regardless of the presence or absence of anxiety and depression symptoms and the type of program. Furthermore, the prevalence of anxiety and depressive symptoms in the patients in this study was very high compared to other studies that, in lower-risk patients, have a prevalence of 20%^45–47^ up to 30–40%^10,46–54^, which could partly explain the results obtained.

Symptoms of anxiety and depression as well as the type of cardiac rehabilitation program influence the bodily pain dimension. Pain in these cases is quite complex due to its heterogeneous nature and can be a lonely experience. Although both programs include the same education and multidisciplinary care components, group dynamics during exercise sessions in the hospital program foster personal connections and social interactions with others whose lives have been affected in the same way^9^. Patients with similar experiences can be a tremendous support and have a greater impact. They feel truly understood. They frequently share fears, concerns, barriers, struggles and coping strategies in a different context compared to the patients in the home-based cardiac rehabilitation program who only visit the hospital once a week. Providing effective and targeted pain support for these patients can be difficult for the family and even for the professionals involved in the cardiac rehabilitation program^55^. Future studies should consider the creation of peer pain support groups in the design of home care programs, especially for those patients who have symptoms of anxiety and depression.

### Study limitations

There are some limitations regarding the sample size of the groups. However, bearing in mind that there are no studies that have analyzed the effect of these programs considering symptoms of anxiety and depression and there were no differences in the percentage of patients with or without symptoms of anxiety and depression between hospital- and home-based cardiac rehabilitation programs, a sample size as small as 10–15 per group is sufficient^56^. Nonetheless, it should be noted that the presence of women in this study was limited despite this being a multicenter study. Unfortunately, this is very common in cardiac rehabilitation studies due to the lower participation rates of women in these programs. Future trials that include underrepresented population, as female and older population, are needed.

Diagnosing anxiety or depression disorder in patients with CVD is difficult given the substantial overlap between the symptoms of anxiety and depression and those of CVD. Indeed during the cardiac rehabilitation program, no patient was diagnosed with anxiety and depression disorder. This study was based on routine screening for symptoms of anxiety and depression and thereby at risk of anxiety and depression. Considering the difficulties in diagnosing anxiety and depression disorders in these patients and the urgent need to optimize the early detection of these symptoms and thereby the prevention of these disorders, the use of simple and brief screening tools such as the Goldberg questionnaire has proven to be very useful in daily clinical practice. Goldberg questionnaire screening patients with symptoms and thereby at risk of anxiety and depression but it isn't a diagnose tool.

On the other hand, the high proportion of patients with symptoms of anxiety and depression compared to other similar studies could be related to differences in the questionnaires used, but it is also possible that these differences are also due to the fact that most of these studies focus on patients with low risk, while our study focuses on patients with coronary heart disease and higher risk.

Finally, although we excluded from the study patients with other comorbidities, social variables or anxiolytic and depression medication have not been included in the analysis.

Despite these limitations, the results of this study have several clinical implications and could improve the models of cardiac rehabilitation in patients with coronary heart disease and moderate risk associated with anxiety and depression symptoms.

## Conclusion

The prevalence of symptoms of anxiety and depression is high in patients with coronary heart disease and moderate risk participating in cardiac rehabilitation programs and has a negative effect on quality of life in these patients, especially on physical and emotional role functioning. Routine screening of these symptoms is needed to improve the design of cardiac rehabilitation programs.

The home-based cardiac rehabilitation program implemented in this study has proved to be effective, especially in patients with symptoms of anxiety and depression, improving quality of life and reducing symptoms of anxiety and depression. Indeed, while the improvement in health transition and functional capacity in both programs was independent of the presence or absence of symptoms of anxiety and depression, improving in the other dimensions of quality of life were significantly greater in those patients with these symptoms, except bodily pain dimension. While the hospital-based cardiac rehabilitation program improved the pain dimension, the home-based cardiac rehabilitation program had a negative effect that was much greater in patients with anxiety and depression. New models of health care should take these factors into consideration to improve the care of these patients.

## Methods

### Eligibility and study design

A complete description of the study design has been published previously^17^. Briefly, a multi-center, randomized controlled clinical trial was designed and is currently ongoing (NCT02796404). Patients recruitment into this clinical trial began in November 2014 and ended in June 2018. The study included patients with stable coronary heart disease who had undergone revascularization by stent angioplasty or coronary bypass surgery with moderate risk according to the clinical practice guidelines of the Spanish Society of Cardiology^19–23^, aged ≤ 75 years, with a good cognitive level, capacity to perform aerobic exercise on a treadmill or cycle ergometer and familiar with the use of a smartphone or tablet. Patients also had to meet at least one of the following inclusion criteria: ventricular ejection fraction dysfunction 40–55%, functional capacity 5–7 METS and/or elevated blood pressure on exertion.

We excluded from the study those patients with malignant arrhythmias, previous myocardial infarctions, exercise-induced ischemia, unstable angina, non-revascularizable disease, poorly controlled hypertension, associated valvular heart disease, pacemaker or ICD-CRT carrier, and any locomotor, neurological or respiratory system condition that impaired the ability to walk for long periods of time.

Patients who met the criteria described above were randomly assigned by an independent investigator before start the intervention to either the control group to perform the standard cardiac rehabilitation program in the hospital, or the experimental group to perform the home-based mixed monitoring program. The participants received information about the study methods and provided their written informed consent.

The study protocol complied with the Declaration of Helsinki and was reviewed and accepted by the Malaga Interprovincial Ethics Committee of the Andalusian Regional Ministry of Health and is registered in the clinical trials register (NCT02796404). The study met the requirements of the Andalusian Health System Order SAS 3470/2009 and with Organic Law 15/1999 of 13 December on the Protection of Personal Data.

### Intervention

Two cardiac rehabilitation programs based on physical training, health education and psychotherapy were carried out, both lasting two months. Patients in the standard cardiac rehabilitation program (control group) attended the hospital three times a week to perform the exercise program (24 sessions) and were encouraged to exercise at home according to the recommendations of the European Society of Cardiology. Patients in the home-based cardiac rehabilitation program with mixed monitoring (experimental group) attended the cardiac rehabilitation unit only once a week, where they performed the same supervised exercise session as the cardiac rehabilitation group. Then they did monitored home exercise using a remote electrocardiographic monitoring device at least two additional days per week, although they were encouraged to exercise every day.

The exercise program prescribed in both cases consisted of 15 min of warm-up followed by 30 min of continuous aerobic exercise at 70% of the heart rate reserve according to the Karvonen formula during the first month and 80% during the second month. The patients completed the exercise session with 15 min of relaxation. Once a week the patients also performed a strength training session comprising one or two series of five repetitions at 20% RM with 2–3 min of recovery between each exercise series.

Both groups received a health education session at the hospital once a week with the aim of improving their knowledge and understanding of topics related to the anatomy and functioning of the heart, cardiovascular risk factors, exercise, medication, diet, erectile dysfunction and return to work. All the patients also attended a weekly group psychotherapy session for support and counseling to reduce the emotional impact of the disease, improve health status and reduce the possibility of a new myocardial infarction, accept the disease and improve quality of life.

### Outcome measures

The following measurements were performed at the start of the study and two months after completion of the cardiac rehabilitation programs.

### Assessment of health-related quality of life

The Medical Outcome Survey Form Short (SF-36) was used to measure patient quality of life. The SF-36 consists of 36 items that evaluate eight dimensions with scores ranging from 0–100: physical function; limitations due to physical problems, bodily pain, social function or role, mental health, limitations due to emotional problems, vitality, energy or fatigue, and general perception of health.

The SF-36 Health Survey, among other general questionnaires such as the Nottingham Health Profile and the Sickness Impact Profile, is one of the most widely used questionnaires to assess the effect of cardiac rehabilitation programs^24,25^. The studies published on the metric characteristics of the Spanish version of the SF-36 provide adequate evidence of its reliability, validity and sensitivity for detecting improvement in health-related quality of life after active intervention^26,27^ in patients with different manifestations of coronary heart disease^24^ (Cronbach’s alpha 0.72–0.94).

### Assessment of anxiety and depression

The Goldberg Questionnaire, or Goldberg Anxiety and Depression Scale, was developed by this author in 1988 from a modified version of the Psychiatric Assessment Schedule^28^ to optimize the procedure to detect patients with symptoms of anxiety and depression. The Spanish version has been validated by Montón et al.^29^. The questionnaire comprises two scales, one for anxiety and one for depression, each with nine items. The responses are dichotomous response (Yes/No) with independent scores for each scale. The cut-off points are ≥ 4 for the anxiety scale, and ≥ 2 for the depression scale. For these values, a sensitivity of 83% and a specificity of 82% and a positive predictive value (95.3%) have been reported. The convergent validity of this questionnaire with other widely used screening instruments such as the Goldberg Health Questionnaire, the Mini International Neuropsychiatric Interview or the Primary Care Evaluation of Mental Disorders is high^30^. Its simplicity, together with its ability to discriminate between anxiety and depression and to provide dimensional information on the severity of each disorder separately, have led to this scale being widely recommended as a screening tool, and a valid and suitable instrument for patient monitoring and follow-up^31^.

### Exercise capacity

A cycle ergometer stress test was performed with continuous 12-lead electrocardiogram monitoring to determine exercise capacity based on METS and exercise time (minutes) using the Bruce protocol. The test ended when the participants reached exhaustion or when signs or symptoms of intolerance appeared.

### Statistical analysis

Descriptive analyses were performed to characterize the sample. The outcomes were described using measures of central tendency and dispersion for the quantitative variables and percentages for the qualitative variables. Fisher's exact test and the unpaired *t*-test or the non-parametric alterative, the Mann–Whitney U test, were used to compare the baseline characteristics of the groups. The Kolmogorov–Smirnov test was used to test the assumption of normality. A nonparametric Wilcoxon paired test and the Friedman test were performed to evaluate the effect of time (before and after intervention), group (anxiety and depression) and program type (hospital- and home-based cardiac rehabilitation programs with mixed monitoring) on changes in health-related quality of life. The level of significance was set at p < 0.05 for all tests.

### Ethics approval

The study protocol complied with the Declaration of Helsinki and was reviewed and approved by the Malaga Provincial Research Ethics Committee of the Ministry of Equality, Health and Social Policies of the Regional Government of Andalucía, Spain. The study meets the requirements of order SAS/3470/2009 of 16 December and with Organic Law 15/1999 of 13 December for the Protection of Data of a personal nature.

### Informed consent

The participants received information about the study methods and gave their written informed consent.

## Data Availability

The datasets generated and/or analysed during the current study are not publicly available due to the decision of the sponsor (Andalusian Public Foundation for Health and Biomedicine Research in Malaga-FIMABIS), but are available from the corresponding author on reasonable request.
